# Hemp as a potential raw material toward a sustainable world: A review

**DOI:** 10.1016/j.heliyon.2022.e08753

**Published:** 2022-01-13

**Authors:** A T M Faiz Ahmed, Md Zahidul Islam, Md Sultan Mahmud, Md Emdad Sarker, Md Reajul Islam

**Affiliations:** Faculty of Textile Engineering, Bangladesh University of Textiles, Dhaka, Bangladesh

**Keywords:** Bio-composites, Biofuel, Hemp paper, Hemp textiles, Hempcrete, Sustainability

## Abstract

Global warming as a result of climate change has become a major concern for people all over the world. It has recently drawn the attention of the entire conscious community, with the fear that if not addressed properly, it will result in the extinction of numerous species around the world. At the same time, it will pose a threat to human health, food security, living environment and standard of living. Thereby, possible solutions are being explored accordingly; regulations have been imposed in places binding green production practices, limiting the emission of CO_2_ and emphasis is given on renewable resources along with the search for alternatives to carbon-positive materials. *Cannabis sativa* L. (hemp) has received a lot of attention because of its multipurpose usability, short production cycle, low capital demand in cultivation, possibility of carbon-negative transformation and easy carbon sequestering material. This paper reviews hemp as a very promising renewable resource including its potential uses in paper, textiles, composites, biofuel, and food industry.

## 1 Introduction

Anthropogenic greenhouse gas emission has emerged as the most dominating factor for climate change [1] which is liable for an increase of nearly 1 °C of global temperature above preindustrial level [2]. As a consequence, it is gradually leading world habitants to extinction by opening vulnerabilities of natural systems. Temperatures exceeding species’ physiological tolerances, changes in precipitation patterns, melting ice caps and rising sea levels, positive impact on harmful species [3], extreme weather events, disease outbreaks, increased incidence of skin cancer [4], shifting wind patterns, and expanding fungal diseases, cropping season changes affecting agricultural yield [5], severe food shortage [2], and negative impact on livestock [6] are just a few of the most feared vulnerabilities.

In recognition of the issue, The United Nations Intergovernmental Panel on Climate Change in 2018 has set a threshold of 1.5 °C temperature increase in average warming above preindustrial level [2], meaning that a rise in global warming beyond this level would make the planet less than suitable for human life. In accordance, scientists advise taking control of greenhouse gas emissions, low carbon economy, development of renewable resources, technological changes, and forestation for stopping global temperature rise [7]. Being aligned with the Paris Agreement, UK and France have already set an aim to “net-zero” emissions by 2050 [8]. As an adaptation, efforts are in place for decreasing fossil energy consumption [9], greater attention has been put on optimal management and use of natural resources [10] along with the development of renewable resources. In 2017, The European Union was able to arrange 17.5% of its consumed energy from renewable resources and aims to reach 32% by 2030 [11].

Global deforestation accounts for approximately 12–15% of anthropogenic greenhouse gas emissions [12]; thereby, forestation is considered one of the most valuable strategies for reducing atmospheric carbon concentration since it works as an essential carbon sink [13]. Enhancement of forest carbon stock has been added to the United Nation's REDD + initiative in 2008; the Bonn Challenge, among the others, is working for awareness and restoration of forest globally, intending to restore 350 million ha of forest by 2030 [14]. Since woody forestation takes a longer time [15], scientists emphasize fast-growing short-rotation forestation as a means of quick carbon sink and source of biomass for fuel [12]. Due to these environmental concerns and balancing regulations, renewable raw materials of natural origin like kenaf, hemp, flax, jute etc., are attracting more attention in different industrial sectors [16] for their competitive physical properties against man-made counterparts and capability of higher carbon sinking.

**Figure 1:**
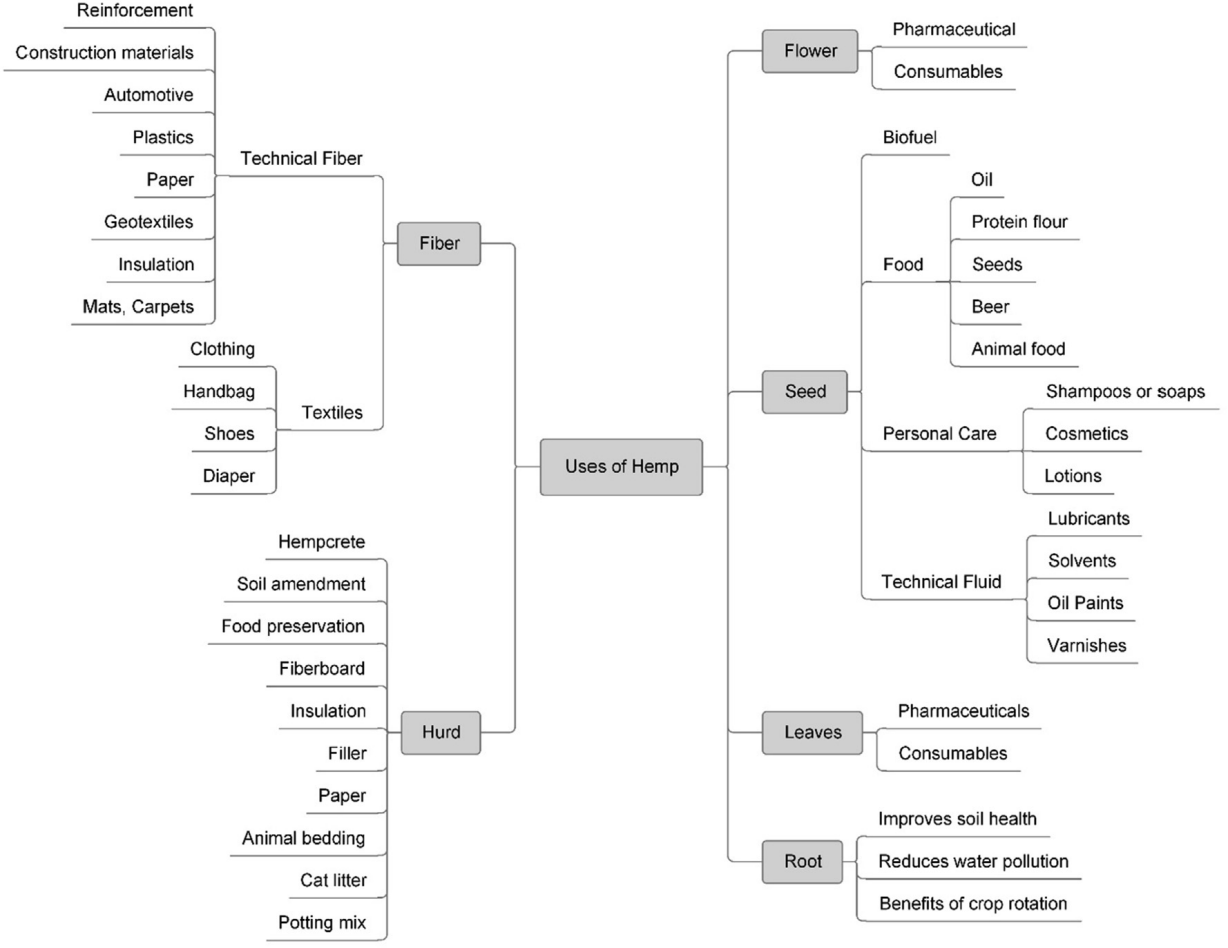
Uses of Hemp for different purposes.

Industrial hemp has emerged as a highly successful commercial crop due to its carbon-sequestering property, higher biomass production, and various end-use products. Researchers believe that it can be successfully used as a cover crop [17] since it can remediate contaminated soils through phytoremediation and can be produced without pesticides. Even hemp residues can act as botanical insecticides or miticides and inhibitors to soil nematodes and pathogenic fungi [18]. It can replenish the soil by killing and displacing other tiny crops or weeds [19] and absorbing heavy metals from soils [20]. Hemp can be used for insulation and acoustic purpose in the building sector, paper industry [16, 21], medicinal purpose, textile industry [20], biofuel, cosmetics industry [22], food and beverage industry [23], and fiber can be used as reinforcement in polymer matrix composites [24] or in bio-composite as a substitute of glass and carbon fiber [25]. The important uses of different parts of the hemp plant in various fields have been shown in Figure 1.

Hemp is classified as industrial hemp containing less than 0.2% Δ^9^-tetrahydrocannabinol (THC) and drug type hemp with greater than 0.2% THC [20, 22]. Although the history of the uses of hemp dates back to 5000–4000 BC [21], prohibition on its cultivation was imposed in many countries in the 20^th^ century [26] because it resembled marijuana [18]. However, after being correctly classified and realizing its environmental and financial benefits, this prohibition is now lifted for industrial hemp. A resurgence in hemp cultivation is seen since the European Union and the US, and other countries, have legalized the cultivation of industrial hemp, and many other countries have reintroduced hemp cultivation with low THC levels [23]. For example, from 2020 Florida Department of Agriculture and Consumer Services is allowing its cultivation with the potential of building a $20–$30 billion industry in the state [27] whereas Europe observed a record amount of hemp cultivation amounting to 33,000 ha in 2016 [20].

The authors in this review have discussed potential uses of hemp in different sectors from the perspective of a green environment with up-to-date knowledge with the belief that other than financial benefits, multipurpose uses of hemp can be a noticeable response to global warming and climate change.

## 2 Cultivation and fiber extraction

Hemp is one of the ancient plants cultivated by humans for textile use. It is believed that hemp cultivation started in western Asia and gradually spread worldwide. Currently, more than 30 countries are involved in the global hemp trade [22] due to its capability of growing in pesticide and herbicide-free environment, noticeable resistance to rodents, fungus and many types of weeds [21], wide geographical range of cultivation, and multipurpose uses. According to FAO Stat (2018), three major hemp-producing countries by production area are Canada (555,853 ha), North Korea (21,247 ha), and France (12,900 ha) [28]. It requires similar soil preparation to other break crops with pH 6.0–7.5 [29]. It grows well on loose, well-drained loam soils with a mean temperature between 16 – 27 °C and high moisture. The final yield depends on sowing density, nitrogen level, harvest time, and it can grow up to 0.31 m in a week [18].

Hemp is a tall, annual crop with a low labor-intensive production process [30] and can be accomplished in a short cropping period (70–90 days). Hemp cultivation has become more appealing to farmers than flax because it has a lower chance of crop failure. It guarantees higher yields (up to12 tons per hectare as cellulose, 20 tons as stem particles and 25 tons as fiber matter per hectare) while also enhancing soil nutrition [31, 32]. The industrial norms for producing this crop are generally guided by organic cultivation to maintain the fiber quality in terms of fineness, strength and color. Hemp fiber cultivation requires about 77.63 percent less cost in fertilization, seeds, field operation, and irrigation costs than cotton, the most recognized natural fiber [33]. It shows many unique properties that differ from other natural fibers by their aseptic properties, high absorbency, protection against UV radiation, and free from allergenic effect [34]. The chemical composition of hemp and some other vegetable fibers has been shown in Table 1, indicating that hemp has higher cellulose, satisfactory hemicellulose and lower content of lignin and pectin, which are advantageous for various processing and uses.

**Table 1:** Chemical composition of vegetable-based natural fibers [35, 36, 37, 38].

Fiber	Cellulose (%)	Hemi cellulose (%)	Lignin (%)	Ash (%)	Pectin (%)	Wax (%)
Abaca	56–63	15–17	7–10	-	-	3
Areca	-	35–64.8	13–24.8	4.4	-	-
Bamboo	26–43	30	21–31	-	-	-
Banana	63–67.6	19	5	-	-	-
Cabuya	68–77	4–8	13	-	-	1.5–2.0
Coir	36–43	0.15–0.25	41–45	-	3–4	-
Cotton	82.7–93.5	5.7	-	-	-	0.6
Flax	71–78.5	18.6–20.6	2.2	1.5	2.2	1.7
Hemp	70.2–74.4	17.9–22.4	3.7–5.7	2.6	0.9	0.8
Henquen	77.6	4–8	13.1	-	-	-
Jute	61–71.5	13.6–20.4	12–13	-	0.2	0.5
Kapok	64	23	13	-	23	-
Kenaf	31–39	15–19	21.5	4.7	-	-
Oat	31–48	27–38	16–19	-	-	-
Pineapple (palf)	70–82	-	5–12	-	-	-
Ramie	68.6–76.2	13.1–16.7	0.6–0.7		1.9	0.3
Rice	28–48	23–28	12–16	-	-	-
Sisal	67–78	10–14.2	8–11	-	10	2
Sugarcane bagasse	45	30	24	-	-	1
Wheat	29–51	26–32	16–21	-	-	-

The fiber extraction process comprises four steps: retting, breaking, scutching and hackling. Field retting (4–6 weeks) and water retting (1–2 weeks) are the most common retting methods [32]. During retting, pectins are broken down, and naturally grown bacteria and fungi bind the fiber together. Then, with the help of fluted rollers, stems are broken down. Next, the stems are separated by beater during scutching, followed by hackling process, and finally combing is done to separate and straighten the fibers.

## 3 Hemp paper

As an ancient raw material, hemp had been used for paper making since 105 AD in China [39]. The technology later disappeared due to emerging of wood-based paper, state-of-the-art technology and market demand [40]. The sourcing, processing, and management of non-woody material like hemp for producing quality paper are now obvious for several reasons. Until the end of the nineteenth century, hemp and wastage of hemp-made household stuff like clothes, ropes, rags and sails had contributed 75–90% of raw materials to global paper production [21]. The first copy of the Bible was written onto hemp papers [39] and the glorious history of hemp paper has been associated with the declaration of independence and the constitution of the US [21].

### 3.1 Demand for non-woody raw material

Papers are said to be sustainable in all formats, but sourcing raw materials require sustainable forestry management [41]. Since the twentieth century, wood has been considered the primary raw material in paper industries. According to Food and Agriculture Organization, 40% of the raw materials for the paper industry come directly from wood [42, 43], and a total of 89–92% is supplied from wood-based materials [40, 44]. The increasing need for paper is continuously putting pressure on forests per capita, whereas regional restrictions are being imposed worldwide by local governments to save forests and the environment. The situation declares the urgency to find new fast-growing raw materials for paper at the place of conventional wood plants [45, 46]. Therefore, from this century, non-woody raw materials like straws, bamboo, kenaf, hemp etc., got considerable interest as raw material for paper-making to reduce the increasing pressure on forest wood resources [47, 48]. In 2010, non-woody fibrous plants contributed 8–11% to global pulp production and the growth rate is estimated to record a 2.5% compound annual growth rate (CAGR) by 2027 [40, 43, 44]. Non-woody raw materials offer easier pulpability, quality bleached pulp, and specialty paper from the selective part of the plants [48].

Hemp yields more biomass than wood, offering even two times more useable fibers than forests [49]. Industrial hemp consists of a maximum of 77% cellulose which is three times more than wood and other agricultural wastes. This indicates a quadruple amount of paper can be produced from hemp against forests grown in the same area [40]. In addition, hemp is a short rotation crop that can be harvested after four months of cultivation, whereas hardwood and softwood plants require 8–12 years and 20–80 years, respectively in rotation cycles [50]. Again, the opportunity to recycle hemp bast fiber-made papers is twice that of wood-based papers [40]. Hemp stalks are composed of long bast fibers and hurds, where the latter is four times more by weight than fiber. Hemp's central woody portion contains 36% cellulose and 27% lignin, whereas bast fiber contains 72% cellulose and 4% lignin [51]. The whole hemp stem contains 47% cellulose and 18% lignin, which is more favorable than pine and birch wood [51]. Hemp stalk contains the highest percentage of cellulose, with the lowest lignin content over almost all non-woody stalks [52, 53]. However, hemp bast fiber secures second for alpha-cellulose after cotton [54]. The lignin and cellulose content in hemp stalks considerably vary among cultivars and growing seasons.

### 3.2 Technical feasibility

The morphology and low lignin content of hemp bast fiber allow easy penetration of processing chemicals resulting faster pulping process [55] with less harsh chemical use [41]. Hemicellulose enhances swelling of the pulp and bonding capacity among the fibers and provides the necessary strength to paper. The hemp core has a higher ability to supply alpha-cellulose to fibrous suspensions and a similar amount of hemicellulose than that of kenaf and hardwood [48]. Hemp fiber exhibited high yield and high tear strength of paper obtained by organosolv pulping process due to having long fibers in pulp [56]. It allows a safer oxygen delignification process with a higher yield than that of birch or pine pulps [45, 51]. The organosolv pulping uses organic solvents to solubilize lignin and hemicellulose, which avoids environmentally harmful sulfur used in conventional pulping techniques, offers high-quality lignin recovery, and easy removal of solvents with less water consumption compared to kraft pulping process.

The organosolv process, which utilizes ethanolamine, produces high-quality hemp core pulp [48]. Moreover, hemp paper processing does not require harmful chlorine bleach; instead, it can use peroxide, which is safer for the environment. In searching for a sustainable pulping process from non-wood feedstock, BioRegional MiniMill technology has been claimed as a promising zero-emission environmentally friendly process for small-scale production [57]. Alkaline pulping of woody hemp core enhances fibrillation and forms inter-fiber bonding [58] which imparts strength in paper and can produce smooth printing grade paper as produced from straw and hardwood pulp [59, 60]. A combination of hemp hurd pulp with hardwood exhibited improved tensile index, bursting strength, softness with identical water absorbency of hand tissue [41]. Blending hemp fiber or woody core with pine [61] and eucalyptus [47] demonstrates higher fiber length in pulp, hence the sufficient paper strength. In most cases, only hemp bast fibers are used for paper-making purposes because of outstanding tearing strength, although lower tensile strength [47]. The successful use of hemp as paper-making material depends on the technical viability of exploitation of both fiber and woody core, not simply one [62] since their differences in morphology and chemical compositions offer suitability for paper-making raw materials. The bleached kraft pulps from hemp stalk were found useful raw material than hemp bast fiber or woody core alone [43]. Organosolv pulping process of whole hemp stalks exhibited the strength between commercial hardwood and softwood pulps [56]. Hemp with core and sheath showed a slightly higher kappa number (an estimation of potassium permanganate required during bleaching) than eucalyptus, indicating that it is more difficult to delignify in kraft cooking process due to lower degree of core fiber polymerization [47]. Strengths are affected due to low hemicellulose content and a lower degree of internal fibrillation [43, 63]. Hemicellulose helps the pulp sheet by strengthening its tensile, bursting, tear strength, specific surface, folding endurance and opacity [48]. The elementary chlorine-free bleaching method with enzyme treatment is not suitable for hemp stalks since it deteriorates the physical properties [52]. It was suggested that separate pulping of fibers and cores followed by blending could yield a pulp with desired properties for multipurpose applications [62]. Hemp fibers are suitable for light and temperature-stable quality office paper [64]. Unlike wood paper, hemp papers survive hundreds of years and do not get into decomposition and yellowing due to aging [21, 40]. Hemp paper's high tear strength and wet strength make it ideal for currency paper, art paper, cigarette paper, tea bags, specialty nonwovens, carbon tissue, grease-proof paper, and other applications [65, 66]. The paper made from hurds can also be used as low-grade printing paper without compromising the quality of the printing surface [67]. Hemp paper showed better oil/air filtration performance than cotton paper in practical application [68].

As a non-wood fiber, hemp also has considerable shortcomings for industrial-scale paper making. Existing paper-making technology is not well capable of handling non-woody crops, and it significantly decreases productivity. In most cases, fibers are separated from the woody core and go through the process separately. The processing technology of hemp woody core and even the separation method of bast fibers must be introduced with state-of-the-art knowledge to reduce costings [49, 62, 67, 69]. Technological advancement is also required to process the whole stalk without separation of fibers and woody core for achieving more yield [48]. Since hemp is an annual crop, and its stalks are very bulky, some difficulties are associated with transportation, storage capacity, and smooth handles [49, 67]. Long-time storage can damage the fibers and cause color change [69]. Pulp quality [69] and yield [70] is related to morphology and chemical composition; they can be affected by harvesting time, geographic location, plant breeding, and even the variation are observed within the biomass [64], which may lead to a possible lack of consistency to paper quality.

The globally produced agricultural residue could produce five times more paper than demand, but technology fails to exploit those in the paper industry [57]. According to the study on different pulping methods of hemp bast fiber, it is found quite acceptable in place of wood from the technical point of view, and even woody cores can also be a promising raw material for paper. Hemp pulps are generally used by mixing with wood pulp, and currently, 100% hemp paper production is not seen [41]. Hemp is speculated to take the opportunity to outvie the other non-wood plants by its yield and growability in moderate or boreal climates. Easy pulping, good quality bleached paper and specialty papers are the key advantages of hemp.

## 4 Hemp composites

Hemp, the second largest grown bast fiber after jute [71], has gained considerable attention as a reinforcement in the polymer matrices in the last decade due to its renewable, biodegradable, and recycling properties. Several composite processing methods have been investigated, including hand lay-up, film stacking, vacuum infusion, manual winding, filament winding, resin transfer molding, pultrusion and injection molding, to name a few. Hemp fiber composites with thermoset, thermoplastic and biodegradable matrices have demonstrated strong mechanical properties due to higher tensile strength of fiber up to 1110 MPa [72]. Table 2 summarizes some important physical and mechanical properties of vegetable-based natural fibers.

**Table 2:** Mechanical properties of vegetable-based natural fibers [73, 74, 75, 76, 77].

Fibers	Density (g/cm^3^)	Length (mm)	Tensile strength (MPa)	Specific tensile strength (MPa/g.cm^−3^)	Young's modulus (GPa)	Specific young's modulus (GPa/g.cm^−3^)	Elongation at break (%)
Abaca	1.5	-	400–980	6.7–20	6.7–20	-	3–10
Alfa	1.4	350	188–350	134–220	22	13–18	1.5–5.8
Bagasse	1.25	-	290	-	17–27.5	-	1.1
Bamboo	1.5–4	-	575	383	11–32	18	2.5–3.7
Banana	-	0.9–4	721.5	534.5	29	22	2
Coir	1.2	20–150	131–220	110–180	2.8–6	3.3–5.2	15–30
Cotton	1.5–1.6	10–60	287–800	190–530	5.5–12.6	3.7–8.4	3–10
Flax	1.5	5–900	700	482.5	27.6–103	18–53	1.2–3.2
Hemp	1.5	5–55	530–1110	360–740	23.5–90	30.5–47	1.6–3
Jute	1.3–1.5	1.5–120	325–800	230	8–78	7.1–39	1.5–2.5
Kapok	-	-	93.3	300	4	12.9	1.2
Kenaf	1.21	2–11	743	-	14.5–53	-	1–2
Pineapple	0.8–1.6	3–9	1020	708.5	71	49.5	14.5
Ramie	1.5	900–1200	400–938	270–620	23	29–85	2–3.8
Sisal	1.3–1.5	900	460–855	317.5–610	9–38	6.7–20	2–2.5

### 4.1 Hemp fiber-reinforced thermoplastic and thermoset composite

Thermoplastic matrix composites perform better over thermoset matrix composites concerning high specific strength, corrosion resistance, cost efficiency, recyclability and design versatility. But the main drawback of natural fiber-reinforced thermoplastic composite is that their processing temperature needs to be kept below 230 °C to protect them from thermal degradation [78]. Hemp fiber begins to degrade at a temperature above 150 °C, hemicellulose and pectin decompose at around 260 °C, while cellulose decomposes at about 360 °C [79]. Hemp fiber-reinforced thermoplastic composites are typically made of polyethylene, polyurethane, or polypropylene matrices. It shows the potential to replace synthetic fiber composites in many lightweight and low-cost applications [78, 80]. The frequently used thermoset matrices for hemp fiber-reinforced composites are epoxy resin, phenolic, vinyl ester, or unsaturated polyester resin. Composites made from hemp fiber with thermoset matrices are creep resistant, solvent resistant, and tough [81]. Wötzel et al. investigated a life cycle study on materials reinforced with hemp fiber against ABS (Acrylonitrile Butadiene styrene) for car parts. Their study revealed that cumulative energy demand for producing hemp composite was half compared to the ABS basic component and hemp composite for an inner lining of cars showed more ecological benefits [82].

### 4.2 Hemp fiber-reinforced green composite

Green composites are referred as bio-composites, the combination of natural fibers with biodegradable polymeric materials. Researchers focused on green composite due to dwindling fossil fuel resources and their negative impacts on environment. The biodegradable polymer matrices such as epoxidized soy oil [83], acrylated epoxidized soybean oil [84], starch-based emulsion [85], cashew nutshell [86], euphorbia oil [87], cellulose acetate [88] and polylactic acid [89] were used to develop hemp fiber-reinforced green composites in number of studies and outstanding mechanical properties for primary structural applications were observed. Though cost and service longevity due to decomposition in nature are associated as drawbacks with green composites [90, 91] further study may open new door to overcoming these barriers.

### 4.3 Performance of hemp composite

Mechanical properties of fiber-reinforced composites strongly depend on fiber length, diameter, orientation, degree of dispersion, aggregate formation and fiber-matrix compatibility [92, 93, 94]. Hemp is susceptible to thermal and oxidative degradation during processing [93]. Another key impediment to manufacturing hemp-reinforced composites is the inability of hydrophilic lignocellulose fibers to adhere to hydrophobic matrices [71, 95]. Due to their polar surface character, natural fiber reinforcement shows lower compatibility with strongly apolar thermoplastic matrices [96]. Again, vapor and void creation during processing due to moisture content [94, 97], photodegradation due to UV radiation [98], and poor resistance to moisture [99] can significantly affect the performance of the composite for outdoor application. A summary of the performance of hemp composite against moisture can be seen in Table 3.

**Table 3:** Susceptibility of hemp composite to moisture.

Fiber fraction	Matrix Type	Pretreatment	Processing technique	Saturated moisture uptake (%)	The slope (k) from M_t_ versus t^1/2^ plot	Diffusion coefficient (D) (10^−9^ m^2^/s)	Ref.
Hemp (5%)	Cyanate ester and benzoxazine	Cyclohexane/ethanol wash + Alkali	Compression molding	2.35	-	5.89	[100]
Hemp (5%)	Polybenzoxazine	Cyclohexane/ethanol wash + Alkali	Compression molding	2.35	0.179	7.12	[92]
Hemp non-woven mat (10%)	Unsaturated polyester	-	Compression molding	3.441	0.102	1.551	[101]
Hemp (10%)	Polybenzoxazine	Cyclohexane/ethanol wash + Alkali	Compression molding	3.45	0.278	7.96	[92]
Hemp (10%)	Cyanate ester and benzoxazine	Cyclohexane/ethanol wash + Alkali	Compression molding	3.52	-	6.81	[100]
Hemp (15%)	Cyanate ester and benzoxazine	Cyclohexane/ethanol wash + Alkali	Compression molding	3.95	-	7.18	[100]
Hemp (15%)	Polybenzoxazine	Cyclohexane/ethanol wash + Alkali	Compression molding	4.25	0.349	8.27	[92]
Hemp non-woven mat (15%)	Unsaturated polyester	-	Compression molding	5.639	0.247	3.618	[101]
Hemp (20%)	Polybenzoxazine	Cyclohexane/ethanol wash + Alkali	Compression molding	4.99	0.419	8.65	[92]
Hemp (20%)	Cyanate ester and benzoxazine	Cyclohexane/ethanol wash + Alkali	Compression molding	5.10	-	8.27	[100]
Hemp non-woven mat (21%)	Unsaturated polyester	-	Compression molding	8.161	0.346	3.841	[101]
Hemp (25%)	Polybenzoxazine	Cyclohexane/ethanol wash + silane	Compression molding	4.47	0.247	9.71	[94]
Hemp (25%)	Polybenzoxazine	Cyclohexane/ethanol wash + Alkali	Compression molding	5.33	0.254	10.26	[94]
Hemp (25%)	Polybenzoxazine	Cyclohexane/ethanol wash	Compression molding	7.06	0.297	11.67	[94]
Hemp non-woven mat (26%)	Unsaturated polyester	-	Compression molding	10.972	0.496	4.367	[101]
Hemp (30%)	Polybenzoxazine	Cyclohexane/ethanol wash + Alkali	Compression molding	6.49	0.481	10.2	[92]

**Figure 2:**
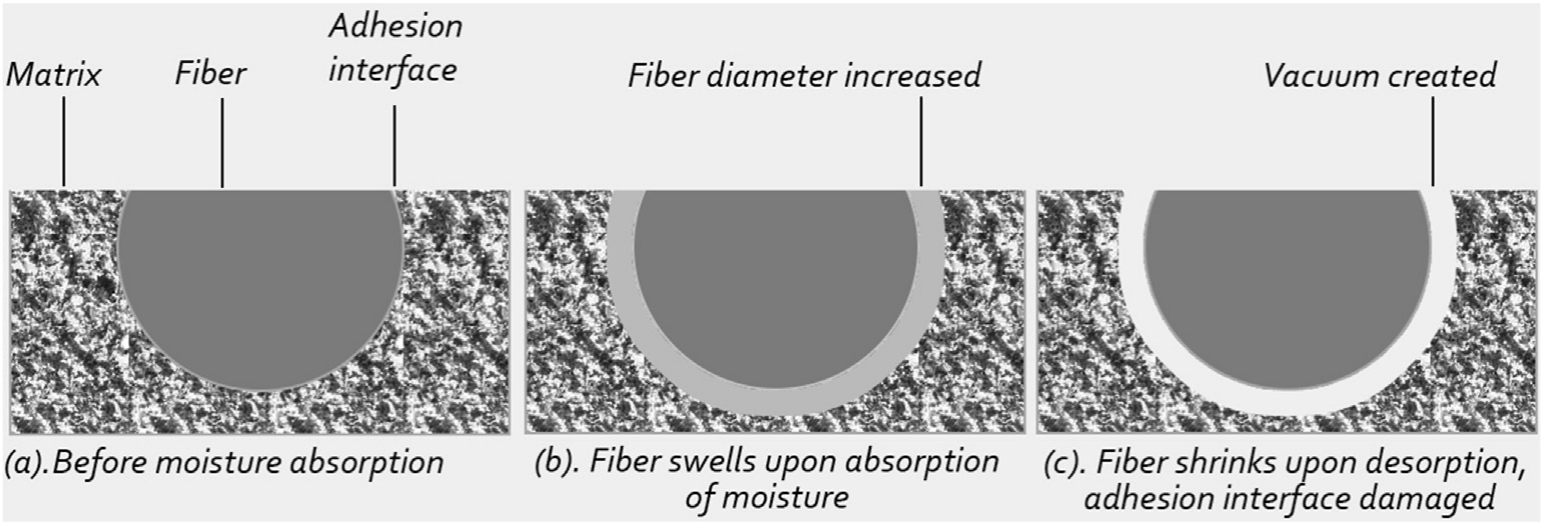
Fiber-matrix adhesion under cyclic absorption and desorption.

Hemp composites absorb moisture, and when dry, the constituent fibers shrink rapidly. Thus interfacial bonding of composites no longer can retain due to cyclic absorption and desorption of moisture and eventually debonding cracks occur inside the structure [102, 103, 104] which allows room for further water penetration [100]. The schematic diagram of the mechanism is illustrated in Figure 2.

Alongside the formation of cracks, moisture uptake also encourages colonial fungal growth that further accelerates the degradation of the composite [95]. Several researchers studied the relationship between fiber fraction and amount of moisture absorption and concluded that for natural fiber, moisture absorption increases with increasing fiber loading [92, 100].

**Figure 3:**
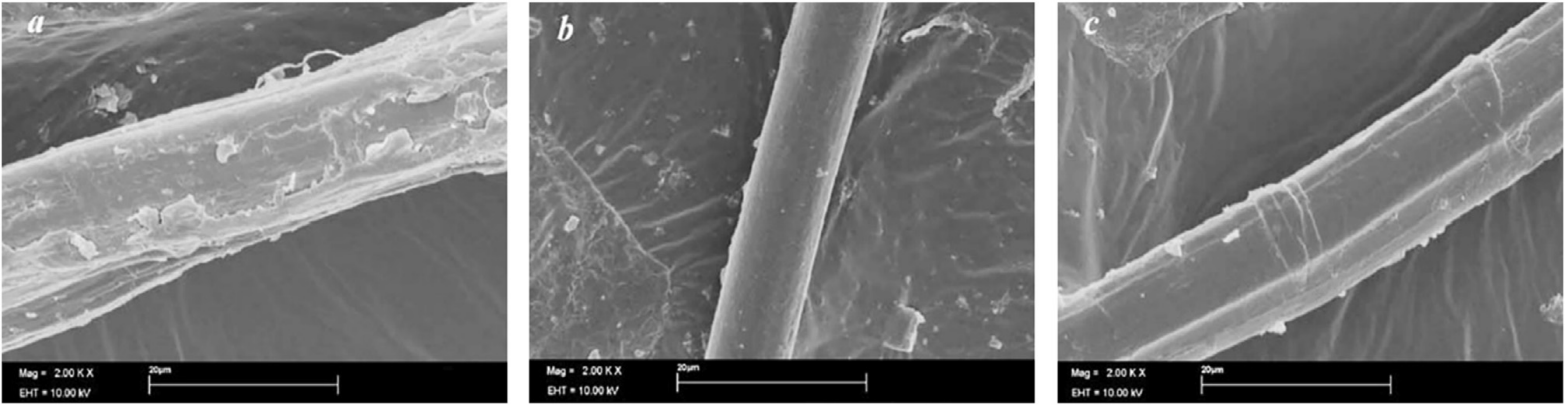
SEM micrographs of untreated and esterified hemp fiber: (a) untreated, (b) acetylated and (c) propionylated (Reprinted with permission from [112]; Copyright (2005) Elsevier).

For solving these inherent limitations, many studies were carried out on physical and chemical modification of natural fibers to enhance surface characteristics and effectiveness as a reinforcement material [99, 105, 106, 107]. Coupling agents were used in some experiments in chemical modification and better compatibility was achieved between cellulose fibers and hydrophobic polymers [108, 109]. Treating hemp fibers with methacrylic anhydride increases interfacial adhesion between hemp and polyester matrices [110]. Mwaikambo and Ansell found that hemp fibers with a 4% & 6% alkalized treatment had the highest modulus and tensile strength [111]. Propionylation and acetylation treatments on hemp fibers resulted in a reduced hydrophilicity but at the same time, decreased crystallinity slightly. The Scanning electron microscope (SEM) results (Figure 3) showed that the esterified materials’ surfaces were smoother than the untreated hemp fibers [112].

Dayo et al. studied the influence of different chemical treatments on hemp composite and recorded the lowest water absorption for silane-treated fiber [94]. The same author also reported a similar result from another experiment where only washed, alkaline treated and silane treated hemp fibers were compared [96]. Among the resins, Polybenzoxazine polymers showed better resistance against moisture absorption with additional advantages like good mechanical and thermal properties [71, 92].

Oza and Lu investigated the effects of silane and NaOH treatment on the thermal and thermomechanical properties of hemp fiber-reinforced high-density polyethylene composites. They observed that thermal stability decreases as fiber loading increases, and treated fiber composites show higher thermal stability than untreated fiber composites. The storage modulus of treated composites was higher than that of untreated composites in dynamic mechanical analysis. Up to 40% fiber loading, the storage modulus value increased, while it dramatically decreased at 50% fiber loading. It was found that the storage modulus of Silane-treated composites was higher than NaOH-treated fiber composites [113]. A similar achievement was also reported by Dayo et al. [94]. A summary of hemp composites' mechanical, thermal and thermomechanical performance can be seen in Tables 4 and 5, 6, respectively.

**Table 4:** Mechanical performance of hemp composites.

Fiber fraction	Matrix Type	Pretreatment	Processing technique	Tensile strength (Mpa)	Young's modulus (Gpa)	Flexural Strength (Mpa)	Flexural Modulus (Gpa)	Impact strength (kJ/m^2^)	Ref.
Hemp (5%)	Cyanate ester and benzoxazine	Cyclohexane/ethanol wash + Alkali	Compression molding	36.58	2.07	-	-	-	[100]
Hemp (10%)	Polybenzoxazine	Cyclohexane/ethanol wash + Alkali	Compression molding	40.21	2.72	-	-	-	[92]
Hemp (10%)	Cyanate ester and benzoxazine	Cyclohexane/ethanol wash + Alkali	Compression molding	41.58	2.45	-	-	-	[100]
Hemp (15%)	Cyanate ester and benzoxazine	Cyclohexane/ethanol wash + Alkali	Compression molding	47.45	2.96	-	-	-	[100]
Hemp (15%)	Polybenzoxazine	Cyclohexane/ethanol wash + Alkali	Compression molding	46.32	3.25	-	-	-	[92]
Hemp (20%)	Thermoplastic starch	Cyclohexane/ethanol wash	Melt processing	4.0	0.182	-	-	-	[114]
Hemp (20%)	Unsaturated Polyester	-	Resin transfer molding	32.9	1.421	54.0	5.02	4.8	[81]
Hemp (20%)	Polybenzoxazine	Cyclohexane/ethanol wash + Alkali	Compression molding	53.26	3.81	-	-	-	[92]
Hemp (20%)	Cyanate ester and benzoxazine	Cyclohexane/ethanol wash + Alkali	Compression molding	55.74	3.47	-	-	-	[100]
Hemp (21.93%)	Epoxy/lignin	-	VARTM	31.15	5.11	86.16	4.18		[115]
Hemp (25%)	Polybenzoxazine	Cyclohexane/ethanol wash	Compression molding	35.2	3.32	112.2	3.96	3.02	[94]
Hemp (25%)	Polybenzoxazine	Cyclohexane/ethanol wash + Alkali	Compression molding	44.8	3.65	113.9	4.16	3.61	[94]
Hemp (25%)	Polybenzoxazine	Cyclohexane/ethanol wash + silane	Compression molding	52.2	4.02	123.2	4.37	4.19	[94]
Hemp (30%)	polylactic acid	Alkaline treatment	Compression molding	53.63	5.60	70.9	7.8	9.7	[116]
Hemp (30%)	Polybenzoxazine	Cyclohexane/ethanol wash + Alkali	Compression molding	62.93	5.07	-	-	-	[92]
Hemp (30%)	Polybenzoxazine	Cyclohexane/ethanol wash + Alkali	Hydraulic hot-press	62.8	5.07	186	5.1	5.73	[117]
Hemp (30%)	Epoxy	-	VARTM	64.0	7.4	91.0	4.7	20.0	[105]
Hemp (35%)	Unsaturated Polyester	-	Resin transfer molding	60.2	1.74	112.9	6.40	14.2	[81]
Hemp (40%)	Polylactic acid	-	Hot press method	44.63	7.4	90	-	-	[89]
Hemp (40 %)	Phenolic	-	Material in steel mould	78	-	105	-	25	[118]
Hemp (50%)	Polybenzoxazine	Cyclohexane/ethanol wash + Alkali	Compression molding	34.46	2.05	-	-	-	[92]

**Table 5:** Thermal performance of hemp composites.

Fiber fraction	Matrix Type	Pretreatment	Processing technique	T_5_ (^0^C)	T_10_ (^0^C)	Y_e_ (800 °C, %)	Ref.
Hemp (5%)	DCBA/BA-a	Cyclohexane/ethanol wash + NaHCO_3_	Compression molding	309.4	357.1	27.9	[93]
Hemp noil (5%)	Polybenzoxazine	Cyclohexane/ethanol wash + Alkali	Compression molding	315	338	28.2	[71]
Hemp (5%)	Polybenzoxazine	Cyclohexane/ethanol wash + Alkali	Hydraulic hot-press	371	406	43.7	[117]
Hemp (10%)	DCBA/BA-a	Cyclohexane/ethanol wash + NaHCO_3_	Compression molding	303.1	350.3	27.8	[93]
Hemp (10%)	Polybenzoxazine	Cyclohexane/ethanol wash + Alkali	Hydraulic hot-press	367	391	43.4	[117]
Hemp noil (10%)	Polybenzoxazine	Cyclohexane/ethanol wash + Alkali	Compression molding	315	337	28.1	[71]
Hemp (15%)	DCBA/BA-a	Cyclohexane/ethanol wash + NaHCO_3_	Compression molding	298.1	346.4	27.8	[93]
Hemp noil (15%)	Polybenzoxazine	Cyclohexane/ethanol wash + Alkali	Compression molding	311	334	28.0	[71]
Hemp noil (20%)	Polybenzoxazine	Cyclohexane/ethanol wash + Alkali	Compression molding	309	332	28.1	[71]
Hemp (20%)	DCBA/BA-a	Cyclohexane/ethanol wash + NaHCO_3_	Compression molding	294.3	342.3	27.8	[93]
Hemp (20%)	Polybenzoxazine	Cyclohexane/ethanol wash + Alkali	Hydraulic hot-press	360	383	42.9	[117]
Hemp noil (25%)	Polybenzoxazine	Cyclohexane/ethanol wash + Alkali	Compression molding	307	330	28.1	[71]
Hemp (25%)	Polybenzoxazine	Cyclohexane/ethanol wash	Compression molding	313	337	28.2	[94]
Hemp (25%)	Polybenzoxazine	Cyclohexane/ethanol wash + Alkali	Compression molding	321	342	28.3	[94]
Hemp (25%)	Polybenzoxazine	Cyclohexane/ethanol wash + silane	Compression molding	324	346	28.5	[94]
Hemp (30%)	Polybenzoxazine	Cyclohexane/ethanol wash + Alkali	Hydraulic hot-press	353	374	42.5	[117]

**Table 6:** Thermomechanical performance of hemp composites.

Fiber fraction	Matrix Type	Pretreatment	Processing technique	E′ (GPa)^a^	Tg (^o^C)^b^	Ref.
Hemp noil (5%)	Polybenzoxazine	Cyclohexane/ethanol wash + Alkali	Compression molding	2.31	175	[71]
Hemp (5%)	Polybenzoxazine	Cyclohexane/ethanol wash + Alkali	Hydraulic hot-press	2.45	193	[117]
Hemp (10%)	Polybenzoxazine	Cyclohexane/ethanol wash + Alkali	Hydraulic hot-press	2.65	196	[117]
Hemp noil (10%)	Polybenzoxazine	Cyclohexane/ethanol wash + Alkali	Compression molding	2.69	177	[71]
Hemp noil (15%)	Polybenzoxazine	Cyclohexane/ethanol wash + Alkali	Compression molding	3.61	180	[71]
Hemp (20%)	Polybenzoxazine	Cyclohexane/ethanol wash + Alkali	Hydraulic hot-press	3.28	203	[117]
Hemp noil (20%)	Polybenzoxazine	Cyclohexane/ethanol wash + Alkali	Compression molding	4.15	181	[71]
Hemp (25%)	Polybenzoxazine	Cyclohexane/ethanol wash	Compression molding	2.98	195	[94]
Hemp (25%)	Polybenzoxazine	Cyclohexane/ethanol wash + Alkali	Compression molding	3.18	211	[94]
Hemp (25%)	Polybenzoxazine	Cyclohexane/ethanol wash + silane	Compression molding	3.41	214	[94]
Hemp noil (25%)	Polybenzoxazine	Cyclohexane/ethanol wash + Alkali	Compression molding	4.26	184	[71]
Hemp (30%)	Polybenzoxazine	Cyclohexane/ethanol wash + Alkali	Hydraulic hot-press	4.15	202	[117]

a Stiffness (E′ at 50 °C).

b Tan δ peak temperature.

### 4.4 Applications of hemp composites

Hemp fibers have significant advantages over synthetic fibers in reinforcing composites and can be used efficiently for a variety of applications because of their high specific strength, low density, low production cost, bio-renewable nature and eco-friendly behavior. The applications of hemp-reinforced composites had been traced in the automotive industry in the 1940s, where Henry Ford produced car components from hemp fiber with soybeans-based bio-matrices [119]. It is predominantly used in the automotive sector to reinforce door panels, passenger rear decks, pillars, and boot linings [120]. Compared to other natural fibers, its uses have remarkably increased in the German and Austrian automotive industries [121]. Due to the higher vibration damping capacity of hemp fibers, researchers also focused on hemp composites in manufacturing sporting goods and musical instruments [122]. Claudio and Marco developed electronic racks for the helicopter by utilizing hemp fabric/epoxy composite materials. The study revealed that this electronic rack from hemp composite was 55.6% lighter than existing steel electronic racks [123]. Hemp fiber reinforced with polycaprolactone composites proved their potential application in fabricating orthotic devices [124]. Hemp chair was developed from hemp yarns with epoxy resin [125], and Xia et al. innovated a hybrid composite from hemp fiber mats and aluminum sheet with epoxy resin which offered excellent electromagnetic interference (EMI) shielding performances [126].

## 5 Hemp plastics

The term "plastic" refers to a material's flexibility or ability to deform into any shape without breaking. Plastic is a carbon chained polymer allowing it to be molded into any shape; that is why they are the most adaptable material [127]. The majority of monomers used to make plastics like ethylene and polypropylene are derived from fossil fuel hydrocarbon. As a result, they are neither biodegradable nor easily decomposable; instead, they accumulate in the landfill and the natural environment [128]. According to literature, roughly 9% of all plastics produced are recyclable, while the remaining 79% end up in landfills and the atmosphere [129]. Petroleum-based plastics and its by-product have a devastating effect on the land, water, and wildlife [21]. For this reason, increasing demand has been started in the world for the usage of high-performance bio-based plastics capable of being environmentally friendly and compensating depleting of petroleum resources [130]. Hemp plastic which is 100% biodegradable, can be a better alternative to synthetic plastic [127]. The cellulose of the hemp plant is rated 60–70%, which can be extracted for making a different range of plastics, including rayon, celluloid and cellophane. While 100% hemp-based plastic is still a rarity, composite bioplastics made from hemp and other plant source are already in use. Though it is by definition a composite, in reference to dimension and end-uses, researchers often use hemp plastics as distinguished terminology.

Researchers have evaluated a range of biopolymers for their usefulness as bio-plastic materials, e.g., cellulose, starch, collagen, casein, plant proteins [131]. Some of the biopolymers for bio-plastics are polybutyrate (PBAT), polycaprolactone (PCL), polylactic acid (PLA) and polyhydroxalkanoate (PHA) [127]. Wheat gluten is one of the most important biopolymers due to its low cost and high content of hydrogen bonds in the film [132]. Wretfors et al. developed short industrial hemp fiber-reinforced wheat gluten plastics and found that hemp fiber-reinforced wheat gluten plastics with 20% fiber content exhibit double tensile strength and ten times young's modulus in comparison to the pure wheat gluten plastics [133]. Wibowo et al. developed hemp fiber-reinforced bioplastics by using cellulose acetate and cellulose butyrate as bio-resin and revealed that hemp fiber-reinforced bioplastics show better mechanical properties than the non-renewable polypropylene-based hemp fiber-reinforced plastics [134]. Hemp-based plastics can be used for packaging and technical purposes. They are particularly suitable because of their strength, lightweight and environmental compatibility.

## 6 Hemp textiles

China, as of now, produces a huge sum of hemp due to its current manufacturing infrastructure, maybe the potential exporter of hemp fiber for textiles [32]. Hemp yarn is usually available in linear density ranging from 2.6 to 54 Nm [135]. Dry spinning method with a shorter drafting zone is preferred for hemp yarn (up to 15.38 Nm) production rather than the wet spinning method [28]. Cotton is generally blended with hemp to reduce its spinning difficulties arising from pectin and lignin of hemp fiber [136]. Fabric from this blended yarn shows better performance in moisture absorption, air permeability, anti-mold and antibacterial property, UV protection and antistatic properties [137]. In a study, hemp noils (comparatively lower length fiber than the standard) were mixed with Uzbekistan cotton in rotor spinning. They obtained good quality yarn with up to 50% hemp fiber content [138]. Hemp also proved its suitability for vortex spinning, and hemp/Tencel air vortex yarns show less hairiness and unevenness than that of the ring and siro spun yarns. Fabric knitted from this type of blended yarn shows significant improvement in lither hand and formability [139]. Stankovic and Bizjak studied the performance of folded yarn in hemp knitted fabric and found noticeable improvement in air and water vapor permeability [140]. In the Hemp/Cotton/Lyocell blended yarn with a higher ratio of hemp, moisture permeability and breathability of knitted fabric increased though at the same time resistance to pilling and abrasion reduced [141]. As the evenness property of hemp blended yarn with the increase of hemp fiber content [142] is usually worsened, researchers suggested that spraying mist in the spinning frame for proper humidification improves the hairiness, evenness, and tenacity of the hemp/cotton blended yarn [143].

Hemp fabrics are available, ranging from 270 to 540 gm.m^−2^ for general to technical use, and fabrics from blended yarn with hemp and wool, silk, or synthetic fiber are more usual [135]. The first production of denim jeans and American flag from hemp is attributed to renowned manufacturer Levi Strauss [65]. Protective clothing is made from a variety of natural fibers, but hemp has some specialty [144]. Union fabric made from hemp yarn (as weft) and cotton yarn (as warp) is more rigid and shows good resistance to flammability, breaking strength, and pilling resistance [145]. Textile products manufactured from natural fibers are comfortable in warm weather though usually deficient in UV absorption, but fabrics from hybrid yarns (hemp as staple fiber; viscose, Polyamide as filament) exhibit better Ultraviolet Protection Factor (UPF) [146]. According to the Chinese Academy of Science, 95% of ultraviolet rays can be blocked by hemp fabric [144].

The alkali and enzyme treatment ensure lignin reduction and improve the surface smoothness and tensile properties of hemp fiber [34]. Moreover, alkali solution increases the dyeing effect in the fabric made from hemp/cotton/polyester blended yarn [147]. Liquid ammonia treatment can improve hemp fabrics’ hand and crease resistance [144]. Fabric treated by sodium hydroxide with flame retardant compound improves the fire-retardant properties and fabric shrinkage [148]. Enzyme treatment [149] makes the hemp fabric softer, smoother, more elastic and highly leveled with minimal strength loss. When hemp is blended with wool, due to the increased amorphous are, it becomes less flammable [144].

Antibacterial textiles have drawn a lot of interest in recent years because of their potential to reduce infection transmission in medical and healthcare environments. Hemp has antibacterial properties that are effective against many pathogenic bacteria [150]. Alkaloids, flavones and saponins are active antibacterial constituents found in hemp fiber [151]. Hemp fiber shows activity against *Escherichia coli*, *Staphylococcus aureus* and *Pseudomonas aeruginosa* [152]. In addition, hemp has anti-mildew properties, and fabric produced from hemp is hypoallergenic [153]. Due to being naturally antifungal and antibacterial, socks from hemp fiber are favored since they keep the feet odor-free [154].

## 7 Hemp edible oil

*Cannabis sativa* L. has been considered an important source of food, fiber and medicine for thousands of years [155]. The hemp seeds are variable in size depending on the cultivar. The actual seeds are enclosed in the pericarp, mostly known as a protective shell. Most of the seed consists of an embryo, mainly the two cotyledons (embryonic leaves), rich in oils, proteins, and carbohydrates, representing the plant's nourishment during germination. After removing the bract, seeds are squashed by applying high pressure for manufacturing oil and a by-product named husk, which is also used for fertilizer and cattle feed [156]. Hemp seed contains 30% protein, 25% carbohydrate and 30% oil [157]. Extraction of the hemp seed oil is carried out by cold pressing methods or applying organic solvent. After the first extraction, approximately 35% oil is left behind in the seed cake. Superior quality oil is obtained from the first extraction process [158]. Cold-pressed seed oil is free from chemical contamination and contains more beneficial components like natural antioxidants that prevent aging-associated diseases like heart diseases, cancer and health problem [159]. The hemp seed oil is a good source of two essential fatty acids: linoleic acid (18:2 omega-6) and alpha-linolenic acid (18:3 omega-3). The omega-6 and omega-3 exist in the ratio of 3:1, comprising the most desirable oil content beneficial for human nutrition [160]. Several studies reported that fatty acid is beneficial for health in preventing cardiovascular diseases [161], capable of reversing scaly skin disorder, inflammation, diabetes [162], and it reduces the risk of cancer and rheumatoid arthritis [163]. The hemp seed oil also contains gamma-linolenic acid that is beneficial for preventing Osteoporosis [162]. The Hemp seed is also a good source of nutrition for birds and fishes as it contains omega-3 and omega-6 in the optimum ratio [164].

## 8 Hemp biofuel

Traditional fossil fuels negatively affect the environment by polluting air and the environment during their manufacturing and use, thereby depleting the ozone level, one of the leading causes of climatic changes and global warming. As fossil fuel is gradually depleting, researchers are looking for sustainable renewable sources to produce biofuel, decreasing the dependency on fossil fuels. Hemp biofuel is considered an effective alternative to decrease the dependence on fossil fuels and reduce greenhouse gas emissions [21, 165]. Nowadays, seeds and biomass are used as energy crops such as wheat grain for ethanol and rapeseed for biodiesel. Accordingly, a variety of energy products can be produced from hemp, such as briquettes or pellets for heat production, biomass for electricity [166], or vehicle fuel e.g., biogas from anaerobic digestion [167]. In terms of the growth stage, biomass increases up to 50 cm/month due to numerous vegetative parts in the hemp plants. It demonstrates the ability to use solar energy and CO_2_ photosynthesis (up to 2.5 Mg ha^−1^), strengthening its position in the group of energy plants that contribute to renewable energy [168]. The digestible concentration of cellulose and hemicellulose is higher than any other crop making it suitable for biofuel. Although hemp biofuel exhibits superior oil quality with higher kinetic viscosity [21], some biofuels have been reported to increase CO_2_ emission if a complete well-to-wheel production pathway is considered [165].

## 9 Hempcrete

The construction sector is a major energy consumer, quantifying nearly 40%, of which 60% is employed for heating and cooling inside the space in developed countries [169]. It contributes almost 32% of global energy demand and is liable for 30% of energy-based CO_2_ emissions [170]. In Europe, the building sector emits nearly one-third of this greenhouse gas [171], whereas the construction material sector is responsible for 10% of worldwide CO_2_ emissions [172]. With a view to intervention, scientists are searching for ecofriendly sustainable carbon-negative materials that would be used as a replacement in full or partial for carbon-positive materials for the construction and building sector. From ancient times, natural resources like plant or animal fibers, straw, etc., have been and still are being tried to include in building materials where hemp is reported to be utilized in the 6th century AD [173].

**Figure 4:**
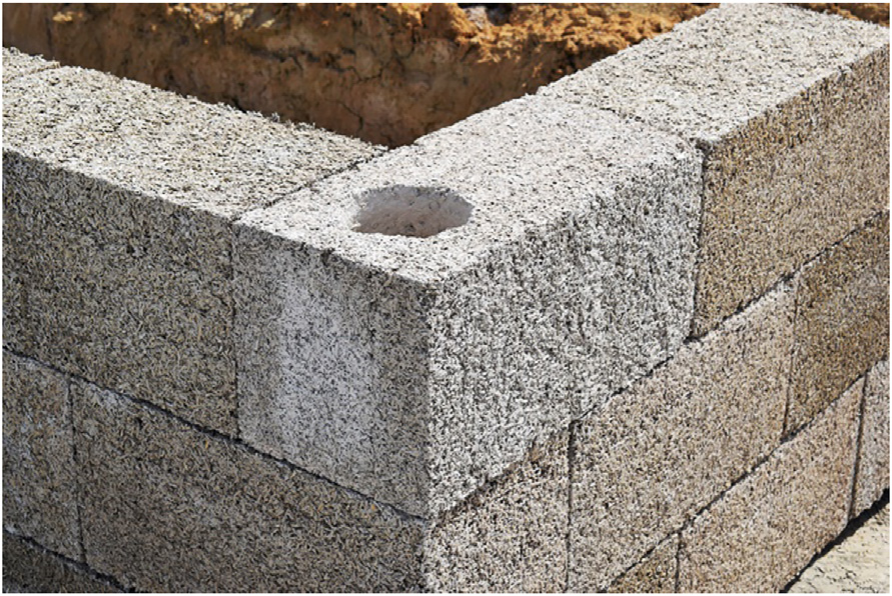
Hempcrete blocks in construction [193].

**Figure 5:**
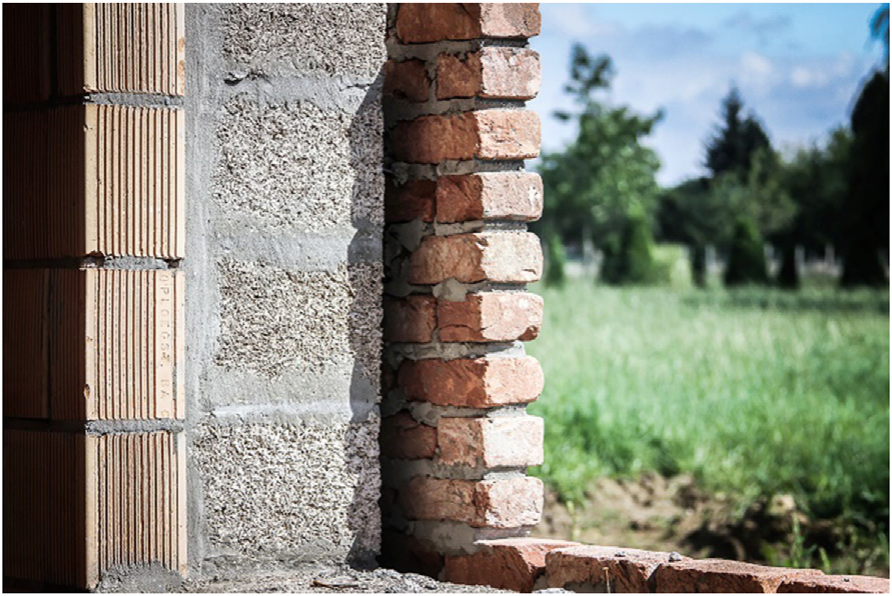
Hempcrete blocks with load bearing blocks [193].

**Figure 6:**
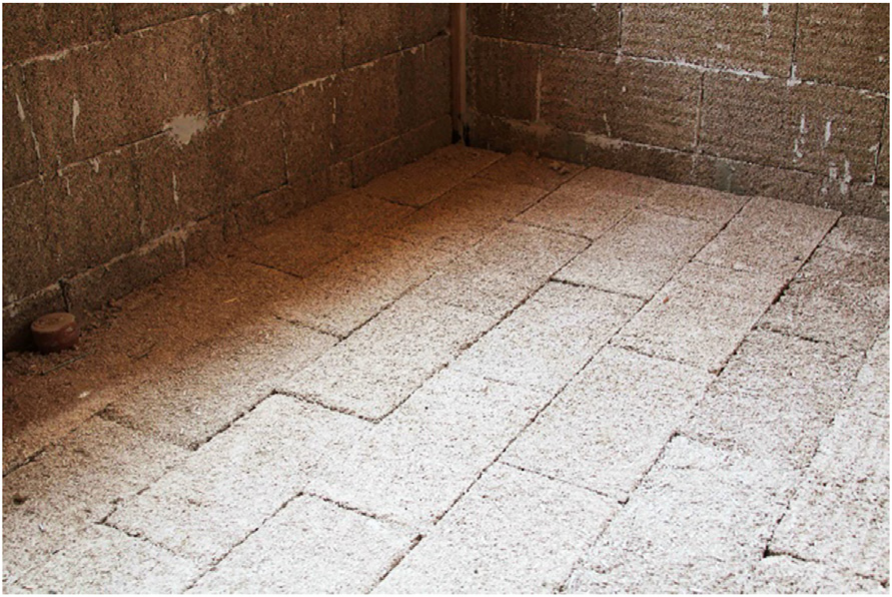
Hempcrete in floor covering [193].

**Figure 7:**
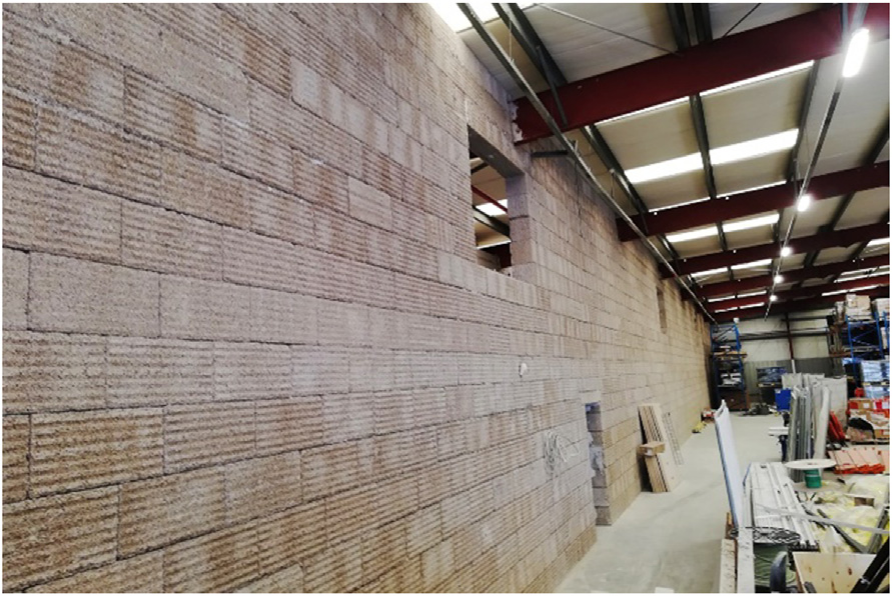
Firewall built with hempcrete [193].

**Figure 8:**
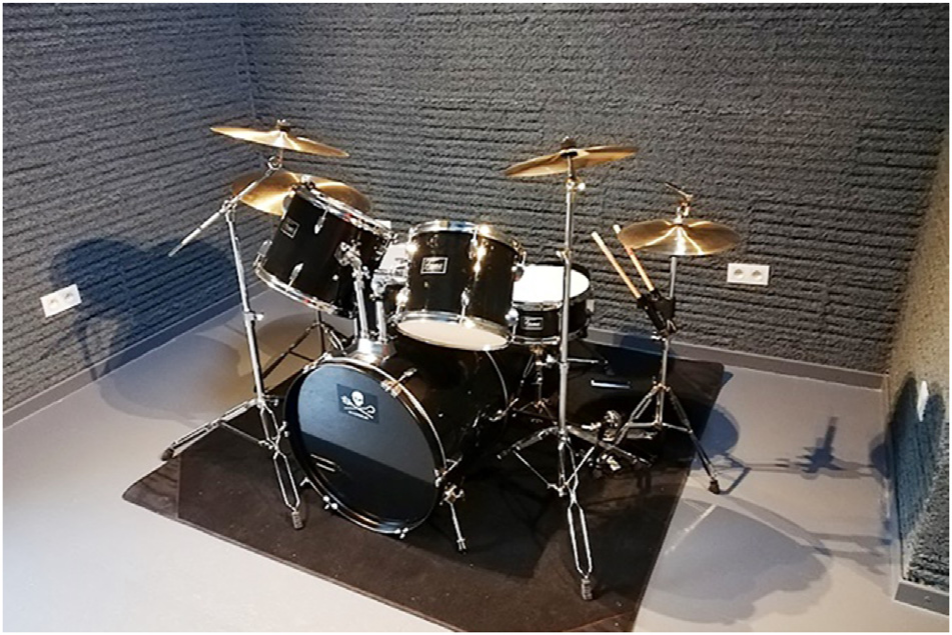
Sound studio with hempcrete [193].

**Figure 9:**
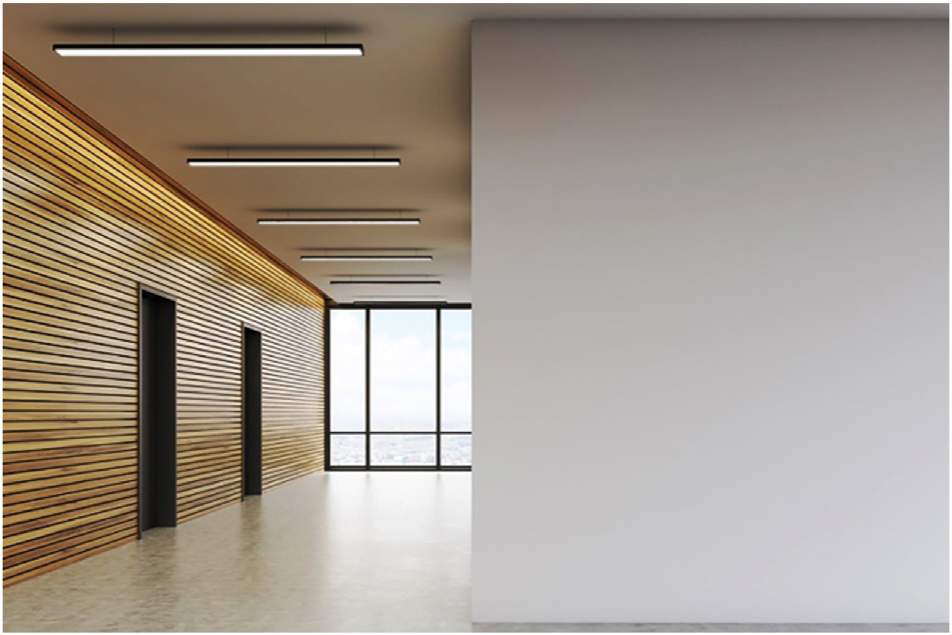
Interior partition with hempcrete blocks [193].

As a natural resource for building material, hemp-lime composite or hempcrete has drawn significant attention recently. It is a composite with mineral binder and plant-based aggregates ground to 5–40 mm long from hemp's shiv or woody core [174]. The properties of the composite depend on binder type, aggregate to binder ratio, size and porosity of the aggregates, and level of compaction [175]. Notable uses of hempcrete started in early 1980 [176], but as shown in Figures 4, 5, 6, 7, 8, 9, hempcrete has proven acceptable as non-load bearing in walls, floors, and roof insulation [177] due to its lightweight, good moisture buffering, thermal insulation, and acoustic properties [175, 178].

### 9.1 Hempcrete preparation

Hempcrete is produced with a mix of ground hurds or shiv, binders in different proportions and water. Cement had been used as the binder [179], but currently, hydrated lime, hydraulic binders and pozzolanic binders like metakaolin are commonly used. Hydrated lime can set through the absorption of CO_2_ during carbonation, but the setting time is extended. Therefore, hydraulic and pozzolanic binders are added [180] to quicken the setting. Varying the proportion within the composition consistent with needs, solid but lightweight and durable [177] hempcrete are often produced ranging density from 200 – 800 kg m^−3^ [178] to be used as wall, floor covering, or roof insulation [179]. Hempcrete can be manufactured by spraying, molding, or manual mixing and tamping [179]. It can be pre-manufactured or freshly made on the construction site. There are some reports of manufacturing it as blocks or hollow blocks or bricks [181]. The compaction due to gravity is negligible though the spraying or projection method is reported to induce higher compaction with minor variation in density [182].

Hemp shiv is a highly hydrophilic porous structure and shows strong capillary action, which ends up in the absorption of water up to nearly five times of its own weight that may often cause a lack of hydration. Therefore for proper slaking of the lime considerable amount of water is required in the mixture, which delays drying and setting time [183]. In contrast, incomplete hydration of binders causes unsatisfactory mechanical strength [184]. In addition to the excessive water, biological compounds especially sugars extracted from the shiv also delay the setting time of the composite and eventually lead to a reduction of mechanical strength of the material [185]. Some researchers suggest that increasing binder hydraulicity may increase early strength development but it doesn't affect ultimate strength regardless of binder type [186].

### 9.2 Mechanical performance

The mechanical performance of hempcrete is relatively modest [176] with very ductile elastic-plastic behavior in compression and tension having a dry density ranging from 200 to 800 kg m^−3^. Most researchers report that this lightweight porous material shows a compressive strength of less than 1 MPa [183] though few pieces of literature claim it to range up to 3.6 MPa after 28 days with a variable Young's modulus [175]. Therefore, its favorable use is as non-loadbearing walls with supporting frames [174, 179, 187] or as finishing plasters with indoor and outdoor insulation properties when density is low, whereas it can also be used as floor or roof insulators with increased density [177]. The causes behind the low mechanical performance of hempcrete have been studied extensively, and the factors identified for inadequate mechanical performance are its high porosity of about 80% in volume, the particle size of the aggregate, voids, too much hydrophilic nature of the aggregate, binder content and setting or curing process [176, 183, 188]. In addition, the level of compaction, flexibility, imperfect particle arrangement [174, 175, 181], and recurring wetting-drying cycles [183] also considerably affect the mechanical strength of hempcrete.

With a view to increasing mechanical performance, quite a number of research have been carried out which studied different aspects of hempcrete. Most of the researchers acknowledge that higher compaction causing the reduction of voids would increase its intended mechanical strength [172, 181, 188], but its thermal insulation [176] and acoustic behavior [189] are compromised. Relative humidity (RH) was also found to influence compressive strength. Curing at different RH was studied with a conclusion that best compressive strength was achieved with curing at 50% RH while curing at 75% RH and 98% RH resulted in worst performance, and low RH (30%) delays the setting of hydraulic binders [188]. Increasing the binder content gradually leads to a lower strain level [188], although it had been suggested for producing panels or building blocks [185]. Mechanical strength of hempcrete varies with the change of binder type; starch-based binders and cement resulted in higher compressive strength than lime binder [190]. Among the Magnesium based binders that show greater compatibility with organic aggregates, magnesium phosphate cement (MPC) showed increased mechanical performance [191]. It was reported that using magnesium oxychloride cement can achieve two times stronger hempcrete without compromising density, thermal conductivity and carbon negativity [185]. Cenosphere binder was also tried as an alternative to lime binders, but although it could retain its integrity at elevated temperature, a noticeable development in mechanical strength wasn't observed [190]. Mineralization of shiv with Al_2_(SO_4_)_3_ and Ca(OH)_2_ indicated achievement of four times compressive strength against non-mineralized hemp shiv with an acceleration of setting and hardening but the carbon negativity of the final product was not reported [192]. Aggregate size can also affect the compressive strength in the long run since smaller particle sizes achieve better coating by binder than bigger particles [188]. Some literature suggested that incorporating flax fiber for hemp-flax concrete increases the density, leading to better mechanical strength and lower shrinkage [183].

### 9.3 Performance against moisture

Building materials can interchange moisture with surroundings, and absorbing, releasing and storing capability of moisture of a material is described by its vapor sorption isotherm. This capability is vital for ensuring human comfort inside the living space [178]. Being highly porous and hydrophilic, hemp shiv can absorb up to 270% water after a few minutes [172] and 400% water of its weight after 48-hour immersion [181]. Hempcrete is quite more permeable than other construction materials and can work as moisture buffering materials [175, 187] due to its fast moisture transport and retention ability and high permeability [194]. This buffering feature offers better control of extreme humidity, decreases vapor condensation, limits micro-organism development and thereby ensures indoor comfort [177, 187]. Due to higher porosity, lower density hempcrete offers more surface area, absorbs and stores more moisture than higher density materials [178]. Binder type can affect capillary action within the material and increasing the hydraulicity of the binder as well as using water retainer can reduce capillary absorption [169, 186]. Hempcrete isn't degradation proof against long-term exposure to rain or extreme humidity [187] and noticeable deformation may occur above 60% moisture content [172], although in the short term exposure, moisture might not propagate deeply into the hempcrete [195]. In a normal situation, shiv shows slow mineralization under the action of lime which makes the composite inert and reduces the risk of rot and mold growth [177]. To prevent the absorption of rain and excessive humidification inside the wall, some researchers suggested using coating or breathable finishing [196] to avoid the possible problem of mold growth over the long run [195].

### 9.4 Performance in thermal insulation

The thermal conductivity (W.m^−1^.K^−1^) of a material is defined as the ability to transfer heat under a temperature difference. It depends on the material's properties, the length of the path that heat flows, and the temperature difference between the two ends. In the case of building materials, this phenomenon is critical since the energy efficiency of a building is greatly affected by the hygrothermal behavior of constituent materials [197]. Air has a low thermal conductivity; therefore, bio-based materials like hempcrete having a highly porous structure result in a lightweight and low thermally conductive material offering better thermal comfort inside the building [186, 187, 195]. The heat transmission of hempcrete ranges from 0.06 to 0.19 W m^−1^.K^−1^ for dry densities between 200 and 840 kg m^−3^ [174]. Due to this low thermal conductivity, hempcrete is suitable to be used in building envelopes [198] to regulate hot waves in summer and reduce heat loss in winter [1] without any additional insulation in masonry works [172]. As mentioned in the previous section, hempcrete has a high moisture permeability and can incorporate a large amount of moisture. It can delay fire spreads by first allowing phase change of capillary water and then hydraulic lime to limestone entangling charred hemp in a brittle configuration [175], resulting in a fire separation media for up to 2 h [193]. It is thermally better than hollow concrete blocks and performs better in summer than Autoclaved Aerated Concrete [196].

The thermal conductivity of hempcrete is affected by formulation, density, water content [169, 183, 186], mold growth, and aging [198]. Lower the density, lower is the thermal conductivity, and better insulation. In fact, the effect of hempcrete's density on thermal performance is greater than that of moisture [197]. Therefore, higher compaction for denser and stronger composite results in lower insulation efficiency [176, 182]. Moisture content affects the thermal performance noticeably, ranging between 0.11 W m^−1^.K^−1^ for dry samples and 0.32 W m^−1^.K^−1^ for samples at 100% RH [175]. Change of binder type does not significantly affect thermal conductivity and specific heat capacity [186, 196], although some researchers noted that increasing binder hydraulicity or the use of water retainers increases conductivity [169]. With the aging, it was observed that moisture storage and water vapor permeability reduced resulting in the increase of thermal conductivity of the composite [198].

### 9.5 Acoustic performance

The property of a material that governs its response to sound waves within a frequency range between 16 to 16,000 Hz is termed as acoustic property. To ensure acoustic comfort by reducing unwanted noise, it is essential that the building materials and design of the building possess good sound insulation properties. Hempcrete inherits high porosity in the structure resulting in a higher sound absorption coefficient which directly affects reverberation time in the room, and sound waves dissipate quickly [175]. The acoustic performance of this composite depends on the aggregate property, binder type, content, and density. Retted hemp was found to perform better than unretted hemp, and hydraulic lime binders contribute better in sound absorption capability than cement binders [175]. Smaller particle size performs better, while higher binder content or denser material strongly reduces the sound absorption capacity of the hempcrete [189].

### 9.6 Carbon sequestering

As mentioned earlier, climate change has emerged as one of the foremost threatening facts for lives on earth. Various types of initiatives are being implemented for the encapsulation of this threat. The European Union has set a goal of reducing greenhouse gas emissions by 40% by 2030 [195]. Construction of buildings and roads consumes nearly half of the raw material and energy across the world [169], and the inside utility services like lighting, heating, and air conditioning emit almost 47% CO_2_ in the UK [199]. Thereby, it can be concluded that this sector is a major contributor to world climate change and requires intensive focus for a review of material design, sourcing, and building design as green building for reducing greenhouse gas emissions.

As an alternative to conventional filling materials, hempcrete can be a better choice for its lighter weight, hygrothermal and acoustic performance, carbon negativity, and natural sink of CO_2_ [169]. It has been reported that 260 mm thick 1 m^2^ hemp-lime wall requires up to 394 MJ of energy and sinks up to 35 kg CO_2_ over a 100-year life span, whereas Portland cement-based equivalent concrete wall requires 560 MJ of energy with an additional release of 52.3 kg of CO_2_ [189]. Therefore, the most potential use of hempcrete in terms of CO_2_ sinking is that its regrowth cycle is in one year, much shorter than forest regrowth for storing carbon over the lifetime of the composite and thereby delaying the emission of greenhouse gas [15].

## 10 Conclusion

Hemp proves competency in the search for new sustainable resources because it is naturally resistant to disease and pests, conserves water, degrades quickly, and produces environmentally friendly industrial products such as biodiesel, bio-concrete, bio-composite, paper, textile, and so on. The hemp biofuel could be an excellent alternative to petroleum-based fuel to produce heat and energy for transport and industrial sectors. Although hemp concretes manifest low load-bearing capacity, they possess excellent mold resistance and good insulation property. In particular cases, it can help decrease the use of cement in building material, which is responsible for the second most CO_2_ emission. The crop would be a new door in the paper industry using its advantage of more yield and more recyclability of hemp paper than wood. The features certainly can slow down the deforestation process.

Rendering its versatility, stiffness, lightweight, and degradation time, hemp plastics can compete with other bioplastics. Hemp composite could be unrivaled in the composite industry for bio-composite demand since the higher strength of hemp fiber imparts outstanding robustness. Furthermore, hemp has proven to be excellent for textile production, and they are now being used in clothing alongside cotton. The modern hemp market has a bright future, as hemp textiles applications and items acquire prominence among producers and purchasers consistently. With the advancement and adaptation of fitting technology, exploitation of the entire physical, chemical and morphological characteristics of hemp can better contribute to a clean, healthy, and sustainable planet.

## Declarations

### Author contribution statement

All authors listed have significantly contributed to the development and the writing of this article.

### Funding statement

This research did not receive any specific grant from funding agencies in the public, commercial, or not-for-profit sectors.

### Data availability statement

Data will be made available on request.

### Declaration of interests statement

The authors declare no conflict of interest.

### Additional information

No additional information is available for this paper.
